# Attenuation of COVID-19-induced cytokine storm in a young male patient with severe respiratory and neurological symptoms

**DOI:** 10.1007/s00508-021-01867-2

**Published:** 2021-04-27

**Authors:** Christian Muschitz, Anita Trummert, Theresa Berent, Norbert Laimer, Lukas Knoblich, Gerd Bodlaj, Alexander Krainer, Christoph Linder, Heinrich Resch

**Affiliations:** 1 Medical Department II—the VINFORCE Study Group, Academic Teaching Hospital of the Medical University of Vienna, St. Vincent Hospital Vienna, Stumpergasse 13, 1060 Vienna, Austria; 2 Department of Neurology, Divine Savior Hospital Vienna, Dornbacher Straße 20–30, 1170 Vienna, Austria; 3 Department of Anesthesiology, St. Vincent Hospital Vienna, Stumpergasse 13, 1170 Vienna, Austria; 4 Medical Faculty of Bone Diseases, Sigmund Freud University, Freudplatz 1, 1170 Vienna, Austria

**Keywords:** COVID-19, Cytokine storm, Baricitinib, Remdesivir, Inflammation

## Abstract

Severe acute respiratory syndrome coronavirus type 2 (SARS-CoV-2), the etiological agent of coronavirus disease 2019 (COVID-19), produces protean manifestations and causes indiscriminate havoc in multiple organ systems. This rapid and vast production of proinflammatory cytokines contributes to a condition termed cytokine storm. A 35-year-old, otherwise healthy, employed, male patient was tested positive for COVID-19. He was admitted to the hospital on disease day 10 due to retarded verbal reactions and progressive delirium. On account of these conditions and the need for noninvasive/invasive ventilation, a combination treatment with baricitinib and remdesivir in conjunction with standard of care was initiated. The cytokine storm was rapidly blocked, leading to a vast pulmonary recovery with retarded recovery of the central nervous system. We conclude that the rapid blockade of the COVID-19-induced cytokine storm should be considered of avail as a principle of careful decision-making for effective recovery.

## Introduction

Severe acute respiratory syndrome coronavirus type 2 (SARS-CoV-2), the etiological agent of coronavirus disease 2019 (COVID-19), produces protean manifestations and causes indiscriminate havoc in multiple organ systems, in particular the lungs, heart, brain, bone, kidneys and vasculature. Cytokines such as interleukin (IL)-1a, IL-1b, IL‑6, and tumor necrosis factor alpha, contribute critically to normal host defences but when produced inappropriately or in excess, they perturb all of the protective functions of the normal endothelium and potentiate pathological processes. This rapid and vast production of proinflammatory cytokines contributes to a condition termed cytokine storm. Induction of IL‑6 production by IL‑1 provides an amplification loop [1].

In a crisis scenario with limited therapeutic resources, patient care often involves management in the ward by means of noninvasive ventilation or high-flow nasal oxygen. Based on clinical judgment or recommendations by the World Health Organization and national authorities, treatment for critically ill patients includes a combination of antiviral (e.g., remdesivir) and anti-inflammatory (e.g., dexamethasone) drugs in an attempt to delay or avoid mechanical ventilation in the intensive care unit.

Janus kinase (JAK) inhibitors, particularly baricitinib, are currently used in the treatment of rheumatoid arthritis. Baricitinib has been proven to interrupt the passage and intracellular assembly of SARS-CoV‑2 into target cells and thereby to disrupt inflammation [2, 3]. Furthermore, in patients with COVID-19, the combination of baricitinib and remdesivir was recently evaluated to have favorable effects on clinical improvements and recovery time [4].

In this short report, we present the results of the aforementioned combination in a young male patient with severe respiratory and neurological symptoms.

## Patient description

A 35-year-old, otherwise healthy, employed, male Caucasian patient was tested positive for COVID-19. Initially asymptomatic, abdominal symptoms emerged. He had two medical visits during self-isolation. Metoclopramide was administered for 5 days orally and once intravenously (disease day 8). On disease day 10 (22 December 2020), he was admitted to the Medical Department II at St. Vincent Hospital in Vienna due to retarded verbal reactions and progressive delirium. The cycle threshold (Ct) value was > 30. Vital signs and oxygen saturation, with the exception of mild sinus tachycardia (110/min), were within normal range and no fever was detected. An acute cranial computed tomography showed a symmetric calcification of basal ganglia, the serum values showed normal calcium metabolism and no signs of hyperparathyroidism, hypoparathyroidism or pseudohypoparathyroidism. The Ct value was above 30, the initial X‑ray of the chest revealed small, symmetrically spotted infiltrates. After neurological consultation, the patient received intravenous crystalloid liquids and oral risperidone 1.0 mg/day (duration 10 days) with the primary diagnosis of an incipient delirium caused by SARS-CoV‑2. Based on the chest X‑ray, a mild oxygen supplementation (1 L/min) was also administered together with anticoagulants and cholecalciferol. The patient had a rapid recovery (within 2h) of all clinical symptoms and remained stable for the following 30 h. Subsequently, on admission day 3 the respiratory and neurological situations deteriorated and the patient developed fever and needed noninvasive oxygen ventilation. Movement disorders and inadequate verbal reactions evolved. Treatment was therefore amplified with dexamethasone (6 mg/day, duration 7 days) and ceftriaxone (2 g/day, duration 8 days). The subsequent blood cultures were negative and no coinfections were detected during the course of treatment. At this point, the serum levels of C‑reactive protein (CRP) increased from initially 50 mg/L to 146 mg/L and of procalcitonin from < 0.05 to 0.13 ng/mL. Due to highly elevated IL‑6 (180 pg/mL) on disease day 12 and after careful evaluation, a combination treatment was initiated with 4 mg oral baricitinib (duration 5 days) and 200 mg intravenous remdesivir (day 1, reduction to 100 mg on the subsequent day, total duration 5 days). Due to an unsatisfactory and continuous decline of peripheral oxygen saturation, the patient received oxygen ventilation up to a limit of 6 L/min. Communication deteriorated with intermittent disorientation and 12h later, with new and ongoing tachypnea and decreasing oxygen saturation values, the patient was transferred to the intermediate care unit (IMCU) for high-flow oxygen supplementation (high-flow oxygen nasal cannula, HFNC. range 40–50 L/min, fraction of inspired oxygen, FiO2, 0.4–0.6). The patient needed HFNC for a total of 75 h and received oxygen supplementation for a consecutive day. A hypokinetic-rigid syndrome based on a COVID-19 encephalopathy was diagnosed at this point in time.

**Fig. 1:**
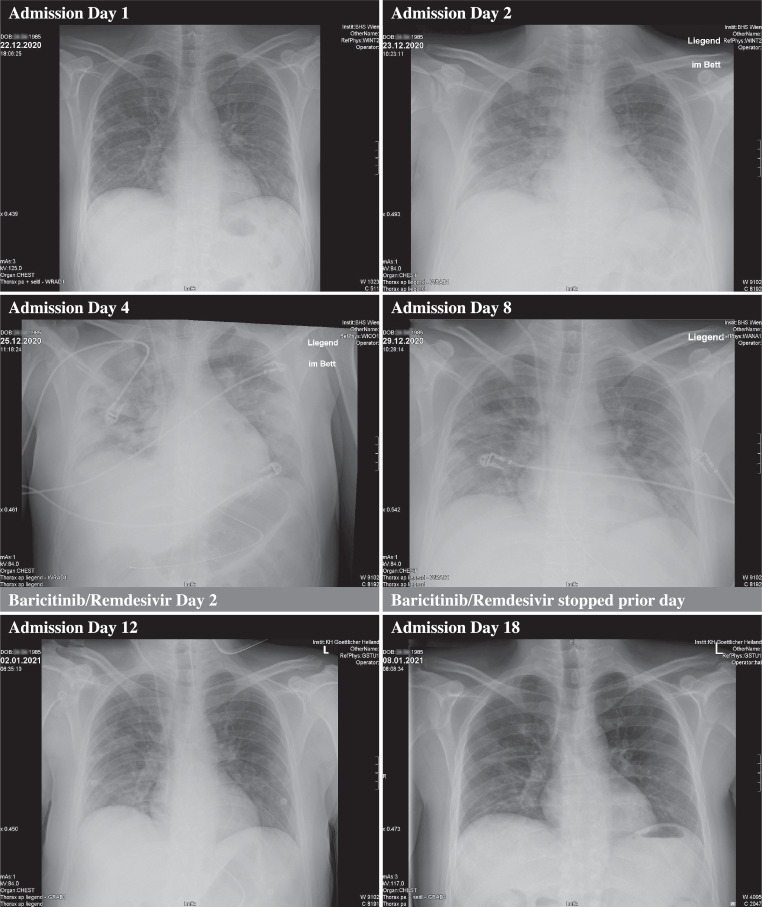
Chest X‑ray images at different time points of treatment

After initiation of the combination treatment with baricitinib and remdesivir in conjunction with dexamethasone, the elevated IL‑6 levels normalized within less than 48 h, the CRP levels were within normal range after 9 days, and consecutive chest X‑rays showed an improvement within 6 days. Inflammatory cytokines remained decreased and within normal range and ferritin remained increased. Bone metabolism showed an isolated increase in osteoclast activity (CTX levels) and the elevated liver enzymes slowly declined (Table 1; Fig. 1).

**Table 1:** Serum values, vital signs and scores at different time points

Hospital admission day Disease day			d1	d2	d3	d4	d5	d6	d7	d8	d9	d10		d18
			d10	d11	d12	d13	d14	d15	d16	d17	d18	d19		d27
			Risperidone											
				Dexamethasone										
					Baricitinib and remdesivir									
Parameter	Range	Unit												
Oxygen flow	–	L/min (L/min/FiO2)	1	4	6	HFNC (50/0.6)	HFNC (40/0.4)	HFNC (40/0.35)	HFNC (30/0.35)	3	2	0	–	0
O2 saturation	(95.0–99.0)	%	98	95	93	98	94	98	100	99	99	98	–	98
SARS-CoV‑2 PCR	–	–	Positive	–	–	–	–	–	–	Negative	–	–	–	–
– N-gene Ct-value	–	–	33.94	–	–	–	–	–	–	–	–	–	–	–
– RdRP gene Ct-value	–	–	34.98	–	–	–	–	–	–	–	–	–	–	–
– E-Gene Ct-value	–	–	33.05	–	–	–	–	–	–	–	–	–	–	–
C‑reactive protein	(< 0.5)	mg/L	51.70	71.70	146.70	153.60	62.00	25.20	13.70	5.30	5.10	–	–	–
Interleukin‑6	(< 7)	pg/mL	12	29	180	35	2	5	4	5	4	–	–	–
Procalcitonin	(< 0.05)	ng/mL	0.04	0.04	0.13	0.16	0.12	0.04	0.03	0.02	0.03	–	–	–
Ferritin	(22–275)	ng/mL	496	1653	1789	1910	1676	1096	1240	804	596	–	–	–
D‑dimer	(< 0.50)	µg/mL	0.60	0.23	0.89	1.07	1.05	1	0.97	1.01	1.06	–	–	–
Lactate	(0.5–2.0)	Mmol/L	1.10	1.21	1.32	0.80	1.10	0.90	0.80	0.60	0.60	–	–	–
paO2	(70–100)	mm Hg	70	86	81	78	63	78	114	106	94	–	–	–
paCO2	(35–46)	mm Hg	35	32	35	36	37	38	38	43	41	–	–	–
Hemoglobin	(13.0–17.5)	g/dL	14.80	13.40	13.80	13.80	120	11.70	11.30	11.90	12.40	–	–	–
White blood cells	(4.00–10.00)	10^9/L	10.16	9.94	16.35	12.12	5.90	8.76	7.93	8.3	12.72	–	–	–
Platelet count	(150–370)	10^9/L	203	208	274	310	289	324	311	361	419	–	–	–
LDH	(< 250)	kU/L	145	187	235	449	348	337	314	196	356	–	–	–
AST	(5–34)	U/L	32	44	32	27	25	31	31	26	47	–	–	–
ALT	(0–55)	U/L	78	64	46	35	35	46	57	71	144	–	–	–
GGT	(0–66)	U/L	88	165	170	139	107	103	97	69	104	–	–	–
Alkaline phosphatase	(43–160)	U/L	65	66	79	121	55	50	45	32	49	–	–	–
Serum creatinine	(0.70–1.30)	mg/dL	0.91	0.67	0.70	0.59	0.56	0.58	0.54	0.40	0.60	–	–	–
Calcium	(2.10–2.58)	Mmol/L	2.26	2.05	2.09	2.07	2.06	2.01	2.01	2.35	2.07	–	–	–
Ionized calcium	(1.17–1.34)	Mmol/L	1.21	1.19	1.18	1.21	1.21	1.23	1.19	1.24	1.22	–	–	–
25-OH vitamin D3	(20.0–70.0)	ng/mL	–	–	–	19.60	–	–	–	–	–	21.20	–	–
CTX	(< 0.584)	ng/mL	–	–	–	0.465	–	–	–	–	–	1.250	–	–
P1NP	(15–59)	µg/L	–	–	–	23.98	–	–	–	–	–	12.56	–	–
iPTH	(15.0–65.0)	pg/mL	–	–	–	13.8–0	–	–	–	–	–	23.70	–	–
UPDRS III score	Max 56	–	–	–	–	–	–	–	–	–	–	49	–	15

*SARS-CoV‑2 PCR* Severe acute respiratory syndrome coronavirus 2 polymerase chain reaction, *Ct value* cycle threshold value, *paO2* partial arterial pressure of oxygen, *paCO2* partial arterial pressure of carbon dioxide, *O2* oxygen, *HFNC* high-flow oxygen nasal cannula, *LDH* lactate dehydrogenase, *AST* aspartate transaminase, *ALT* alanine transaminase, *GGT* gamma glutamyl transferase, *CTX* carboxy-terminal collagen crosslinks, *P1NP* procollagen type 1 N‑terminal propeptide, *iPTH* intact parathyroid hormone, *UPDRS* unified Parkinson’s disease rating scale III, *FiO2* N‑gene, E‑gene RdRP gene

After 11 days at the Medical Department II (including 5 days at the IMCU), the patient was transferred to the Department of Neurology at the partner Hospital of the Divine Savior in Vienna, Austria for further treatment work-up and treatment of ongoing extrapyramidal symptoms. On admission the patient presented with a pronounced hypomimia, rigidity, bradykinesis, tremor and a mild tetraparesis of the extremities as well as dysarthria and dysphagia, resulting in a unified Parkinson’s disease rating scale (UPDRS) III score of 49 (out of possible 56). Risperidone was terminated 2 days before and the family already reported some improvement of speech. Magnetic resonance imaging (MRI) of the brain, a lumbar puncture and an electroencephalogram (EEG) were performed. The MRI revealed no structural alteration of the brain apart from the previously shown basal ganglia calcifications. All cerebrospinal fluid parameters showed no signs of viral or bacterial disease or inflammation, the EEG was normal. Based on the interdisciplinary treatment, the patient recovered rapidly. The initially started levodopa (L-DOPA) treatment (maximum 50 mg tid) was tapered without negative effect. After 14 days under neurological treatment and intensive physiotherapy he was fully orientated, ambulating freely and had normal vital signs and serum values. Slight movement disorders are still detectable, but minor compared to the severity of symptoms at the time point at the admission to the hospital. The UPDRS III score improved to 15 and the patient was discharged from the hospital. Rehabilitation and muscle perseverance training is needed. One month after the first symptoms of the COVID-19 infection a slight reduction in retentiveness is reported by the family.

## Discussion

We report a case of a young patient with COVID-19-associated, initially mild abdominal symptoms and progressive severe respiratory and neurological symptoms induced by a cytokine storm. Proinflammatory cytokines conspire to elicit from endothelial cells a change from their homeostatic functions to those that can contribute to thrombosis and local tissue injury [1]. In line with WHO recommendations, the standard of care in symptomatic patients includes oxygen supplementation and dexamethasone. The deterioration of clinical symptoms and serological findings demands a therapeutic boost on account of the pathophysiology of this novel disease. Based on recently published data from a double-blind, placebo-controlled trial with a comparable study population, this young and otherwise healthy patient received an additional combination of baricitinib and remdesivir [4]. The administered and recently published combination treatment was safe and immediately lowered elevated levels of IL‑6 and other inflammatory cytokines. It probably reduced the duration at the IMCU with HFNC and led to the patient’s recovery with reduction symptoms of the affected organ systems.

Serological, radiological and clinical findings suggest the need for an individual and targeted approach. The decision of treatment enhancement beyond dexamethasone and oxygen with different RCT-proven approaches to reduce the cytokine storm was based on the patient’s rapid pulmonary and neurological worsening in an attempt to avert the use of mechanical ventilation.

The potential role of baricitinib in the treatment of COVID-19 is based on artificial intelligence algorithms. A potent JAK 1/2 inhibitor would be able to directly mitigate the inflammatory response/cytokine storm triggered by the COVID-19 infection. This drug has a high affinity for protein kinases directly involved in the endocytosis of coronaviruses [5, 6]. Disease severity is marked by hyperinflammation and cytokine storm syndromes driven by IL‑6 expression through the JAK signal transducers and activators of transcription pathway [7].

Despite concerns about immunosuppression, secondary infections, or increased risk of thrombosis, the administration of baricitinib in conjunction with dexamethasone or antiviral agents in clinical trials has not been associated with a significantly higher incidence of adverse events. In contrast, the JAK inhibitor baricitinib, with a half-life of 12 h in combination with remdesivir, exerts beneficial effects in terms of the time of oxygen supplementation and the time to recovery. These effects were also observed in this patient and corroborated by numerous serological, radiological and neurological investigations during treatment [4–7].

Besides the pulmonary affection, the patient had also developed delirium and an extrapyramidal syndrome (EPS). Neurological symptoms are common in COVID-19, including anosmia and ageusia, non-specific symptoms, such as dizziness and headache, and severe conditions, such as ischemic stroke, hemorrhagic and/or reversible encephalopathy syndrome with epileptic seizures. Extrapyramidal signs in patients with encephalopathy have been reported. Ischemic and hemorrhagic strokes seem to have a higher incidence in patients with a severe COVID-19 course of disease. The two major competing hypotheses are based on neurotropism and direct invasion of SARS-CoV‑2 into the central nervous system (CNS), and indirect mechanisms mediated by the cytokine storm induced by systemic SARS-CoV‑2 infection. Post-mortem examinations of CNS in this patient population have demonstrated mild neuropathological changes, with pronounced neuroinflammatory changes in the brainstem being the most common finding [8].

In this context it remains unclear if risperidone, which was started due to the delirium contributed to the EPS on the basis of the bilateral calcification of the basal ganglia or if the EPS was part of an encephalopathy syndrome due to COVID-19 infection. To date, evidence in the literature is scarce, although the use of this antipsychotic drug has been proven to be efficacious in this specific context [9, 12]. The dopamine agonist metoclopramide, which was administered prior to hospital admission, is able to intensify extrapyramidal symptoms in patients with Parkinson’s disease. While no adverse events in COVID-19 patients have been published to date, metoclopramide was recently even identified as a protective factor [10].

The SARS-CoV‑2 also causes an activation of nuclear factor kappa-light-chain-enhancer of activated B‑cells (NF-κB) with subsequent bone resorption through direct activation of the receptor activator of NF-κB ligand (RANK-RANKL) system. Recent data suggest that this disease has the potential to act directly on bone resorption units with unfavorable long-term effects on bone metabolism (uncoupling) and a possibly increased risk of fragility fractures [11].

A multifactorial therapeutic approach with immunomodulatory drugs, based on clinical findings and trials, may help to achieve therapeutic goals. We conclude that the rapid blockade of the COVID-19-induced cytokine storm should be considered of avail as a principle of careful decision-making for effective recovery while requiring long-term surveillance of COVID-19 patients.
